# CT-guided preoperative localization of nonpalpable pulmonary lesions with a specifically designed device: evaluation of safety and efficacy

**DOI:** 10.31744/einstein_journal/2025AO1622

**Published:** 2025-09-08

**Authors:** Rayssa Araruna Bezerra de Melo, Demian Jungklaus Travesso, Paula Nicole Vieira Pinto, Marcos Vinicius Amaro Gomes, Joaquim Mauricio Motta-Leal, Fabricio Prospero Machado

**Affiliations:** 1 Prevent Senior São Paulo SP Brazil Prevent Senior, São Paulo, SP, Brazil.; 2 A. C. Camargo Cancer Center São Paulo SP Brazil A. C. Camargo Cancer Center, São Paulo, SP, Brazil.; 3 Universidade de São Paulo Instituto do Câncer do Estado de São Paulo "Octavio Frias de Oliveira" Hospital das Clínicas São Paulo SP Brazil Instituto do Câncer do Estado de São Paulo "Octavio Frias de Oliveira", Hospital das Clínicas, Faculdade de Medicina, Universidade de São Paulo, São Paulo, SP, Brazil.

**Keywords:** Preoperative period, Pulmonary nodule, Neoplasms, Tomography, x-ray computed, Video-assisted surgery, Thoracoscopy, Thoracic surgery, video-assisted

## Abstract

**Objective:**

This study aimed to evaluate the percutaneous preoperative localization of lung masses suspected to be nonpalpable with a wedge-shaped wire (Somatex^®^ Lung Marker System).

**Methods:**

Patients underwent CT-guided lung mass localization with the Somatex^®^ Lung Marker System prior to resection of pulmonary lesions by video-assisted thoracoscopy. The characteristics of the lung masses, complication profiles, histological analysis, and surgical success were reviewed.

**Results:**

Forty lung masses were percutaneously localized preoperatively in 38 patients. Eight patients did not have malignancies. Major complications were not observed. All lung masses were fully resected after preoperative localization.

**Conclusion:**

The findings support the feasibility and safety of the Somatex^®^ Lung Marker System for the preoperative localization of lung lesions.

## Highlights

■This method enables surgeons to visually identify the lesion pathway without intraoperative imaging, enhancing precision and workflow.

■A practical, cost-effective alternative to high-end technologies such as electromagnetic navigation bronchoscopy and hybrid operating rooms.

■Metallic wires offer excellent intraoperative visibility on the lung surface, supporting their clinical utility.

■All nonpalpable nodules were successfully resected, with no complications related to the preoperative localization procedure.

■CT-guided preoperative localization of nonpalpable pulmonary lesions using a purpose-built device demonstrates both safety and efficacy.

## In Brief

Lung cancer screening has led to the earlier detection of small and nonpalpable pulmonary nodules, increasing the demand for accurate preoperative localization techniques. Among recent innovations, computed tomography-guided placement of a spiral-edged wire marker offers a minimally invasive solution for marking nonpalpable lesions before thoracoscopic resection.

## Graphical abstract

**Figure:**
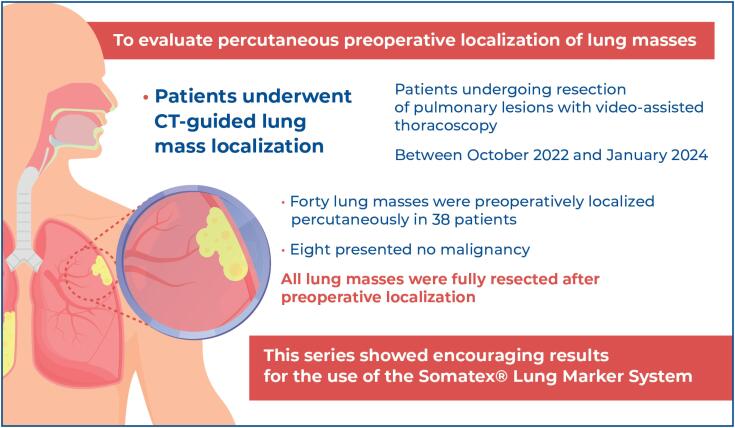

## INTRODUCTION

Implementation of lung cancer screening programs has unequivocally demonstrated a commendable reduction in mortality by up to 20%. These meticulously crafted initiatives are poised to significantly augment the early diagnosis of diminutive and non-solid lesions, reflecting a pivotal shift toward a more effective management of lung cancer.^(1)^ The utilization of pretherapeutic metal markers has become entrenched within mastology and radiotherapy, illustrating the broader application of localization techniques in surgical contexts where lesions elude palpation. This is particularly relevant in cases of pulmonary opacities or diminutive musculoskeletal lesions, where the risk of non-localization during procedures poses a significant challenge.^(2,3)^

Recent advancements have underscored the necessity for developing a dedicated device tailored to address the nuances of lung lesions. This is critical for improving procedural efficacy and surgical outcomes.^(4,5)^

Some new localization methods, including electromagnetic navigation bronchoscopy (ENB) and hybrid operating rooms (HORs), represent significant advancements in the field. ENB localization using patent blue vital dye before thoracoscopy for lung resection offers a new and effective approach for localizing small, deep-seated, and nonpalpable pulmonary lesions, providing an alternative to CT-guided localization.^(6)^ Similarly, the advent of HORs has enabled the simultaneous single-stage localization and removal of lesions, presenting an innovative solution to traditional two-stage approaches and associated complications, such as wire dislodgement and pneumothorax.^(6,7)^ However, high cost and low availability are important limitations of these methods.

Among the innovations in the field, the spiral-edged wire lung marker system has shown promise for preoperative CT-guided localization of small nonpalpable lung lesions, offering a nuanced approach to enhance surgical precision. The percutaneously placed wire marks the trajectory from the skin surface to the nonpalpable nodule, allowing the surgeon to locate the target area solely through visual inspection without auxiliary imaging tools. A possible disadvantage is the need to coordinate the localization procedure with the surgical resection, as they are separate procedures usually performed by different physicians.^(8)^Other potential drawbacks include dislodgement of the fiducial marker and associated pneumothorax.^(8)^ A short interval between nodule localization and surgical resection is recommended, with intervals in most reports ranging from 1 to 24 h.^(5,9)^

This study aimed to evaluate the preoperative CT-guided localization of small nonpalpable lung lesions using a spiral-edged wire lung marker system, integrating recent advancements to demonstrate the importance of precision in surgical interventions. By examining the effectiveness and safety of this method, we intend to optimize the surgical outcomes of patients undergoing resection of pulmonary lesions using video-assisted thoracoscopy (VATS), highlighting the pivotal role of technological innovation in enhancing patient care.

## OBJECTIVE

To evaluate preoperative CT-guided localization of small nonpalpable lung lesions using a spiral-edged wire lung marker system.

## METHODS

This study included patients who underwent resection of pulmonary lesions using VATS and preoperative localization with a spiral-edged wire marker between October 2022 and January 2024.

The thoracic surgery team indicated the localization of the preoperative lung nodules. Traditional indications included unpalpable nodules (ground-glass opacities), small subpleural nodules (less than 10 mm), or nodules that were deeply located at more than 10 mm from the visceral pleura.

**Figure 1:**
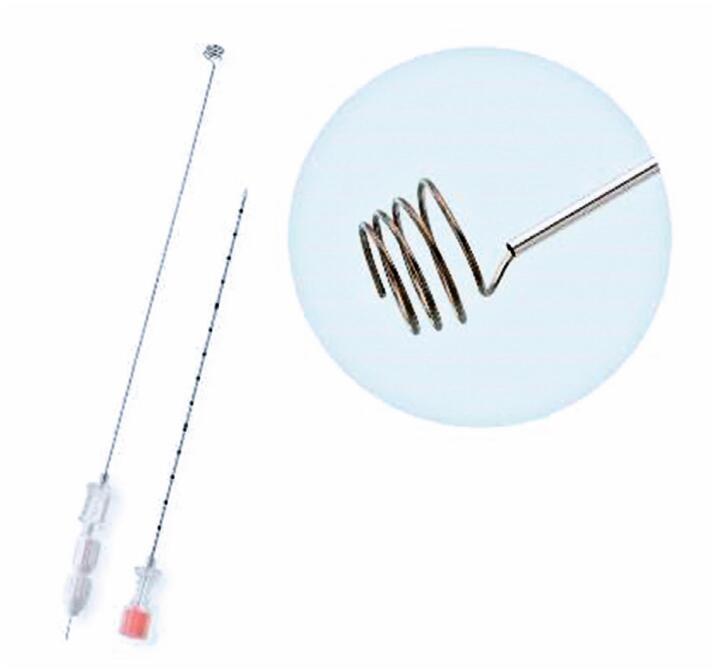
Components of the Somatex Lung Marker system

**Figure 2:**
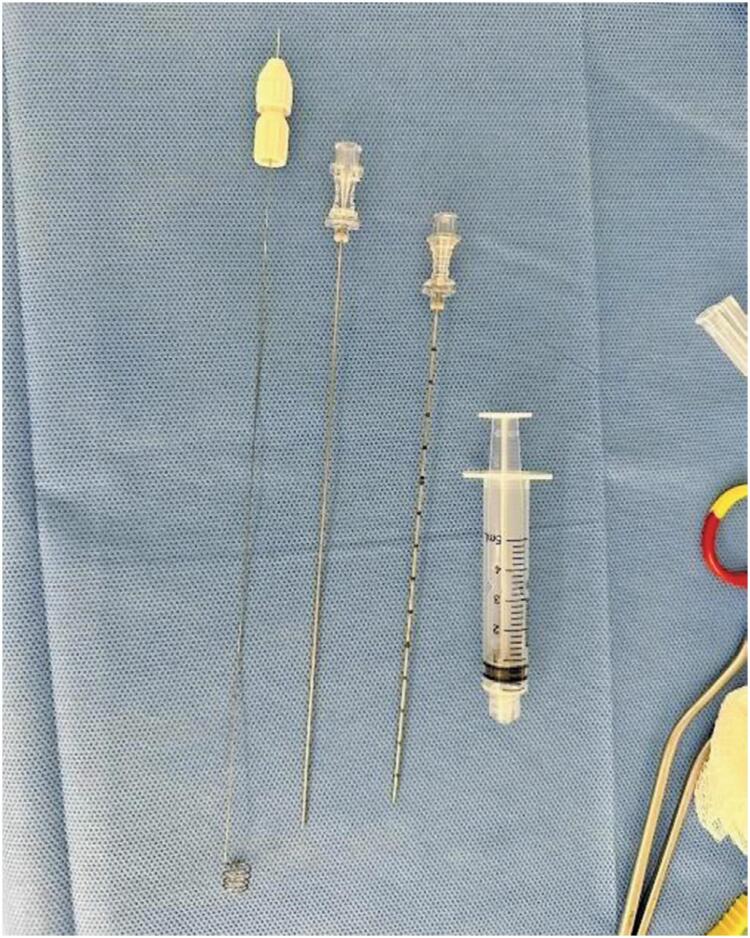
Separated components of the Somatex Lung Marker system, from left to right: an 18-gauge coaxial needle that accesses the lung nodule, a coaxial needle containing the preloaded wire, and the wire with its coiled distal end

The Somatex^®^ Lung Marker System (Somatex Medical Technologies Rheinstrasse 7d - Teltow. Germany) consists of a coaxial 18-gauge needle and a thinner needle loaded with a metallic wire ending in a coil-shaped tip (Figures 1 and 2). This device is specifically designed for the preoperative localization of lung nodules.

The lesion size, pulmonary lobe, localization rate, complications, and histological results were assessed.

Nodule localization was performed under local anesthesia or light sedation in a CT suite using CT imaging guidance (GoldSeal Optima CT 660, 64-slice; GE Healthcare, California, US).

## RESULTS

Forty lung nodules from 38 patients were surgically resected. The patients’ ages ranged from 59 to 81 years (mean 69.1). Percutaneously marked lesions varied in size from 0.6 to 2.1cm (mean 1.2cm), with 15 lesions measuring 1.0cm or less. Lesion size, localization, and histology are shown in table 1. Histological evaluation revealed neoplasia-free margins across all cases.

**Table 1:** Analyzed cases, with patient and lung mass characterizations

Variable		Value
Patients		38
Procedure (lung nodules)		40
Mean age (years)		69.1 (range: 53-86)
Mean nodule size (mm)		12 (range: 6-21)
Lung localization, n (%)		
	RUL	15 (37.5)
	RML	2 (5)
	RLL	4 (10)
	LUL	12 (30)
	LLL	7 (17.5)
Histological findings		
	Adenocarcinoma	31
	Atypical adenomatous hyperplasia	1
	No malignancy detected	8

1 RUL: right upper lobe; RML: right middle lobe; RLL: right lower lobe; LUL: left upper lobe; LLL: left lower lobe.

**Figure 3:**
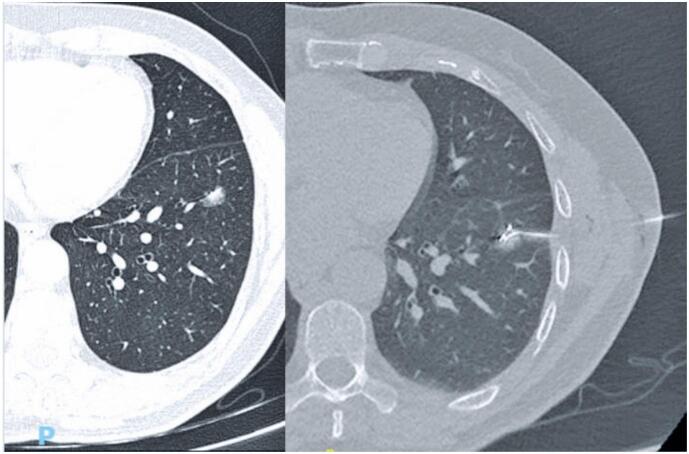
66-year-old female patient with previously resected lung cancer. Follow-up CT shows a new small nodule in the left lower lobe. The lesion was preoperatively marked with the metallic spiral wire and resected. Pathology confirmed invasive lepidic adenocarcinoma

**Figure 4:**
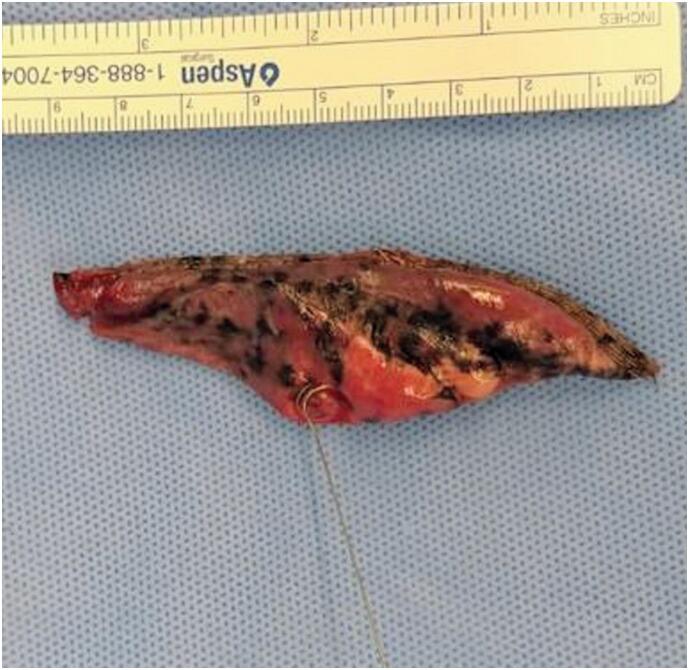
Cuneiform pulmonary resection showing the location of the spiral wire

In 39 nodules, the coil was placed within or at the edge of the lesion; in one case, it was located outside the mass. This did not affect the surgical outcome, as the surgeon was able to trace the needle track into the lung parenchyma. Figures 3 and 4 present CT images of the procedure and macroscopy of the hook wire in position after resection.

Eight patients presented with minor complications, including three laminar pneumothoraces and five perilesional alveolar hemorrhages without clinical repercussions or the need for additional procedures (Grade 1: CIRSE Classification System for Complications).^(10)^

All nodules were located and resected, representing a 100% success rate using CT-guided localization.

## DISCUSSION

The nonpalpable nature of small and subsolid pulmonary nodules poses a challenge for lung surgery, creating the need for a strategic approach to preoperative localization.^(11)^ Various materials, especially metallic wires, have been explored for this purpose.

Alternative materials, including cyanoacrylate, technetium-99 macroaggregated albumin, methylene blue injection, metallic surgical clips, fiducial markers, and coils, offer safe options. Radioguided localization, albeit productive, mandates the deployment of radiotracers, radioprotective equipment, and proficiently trained teams.^(4,5)^

Challenges inherent to specific materials, such as the rapid diffusion of methylene blue into the surrounding tissues or the potential vascular embolization risk associated with cyanoacrylate injection, underscore the nuanced decision-making required to choose the appropriate methodology.^(12)^

CT-guided localization has the advantage of being reproducible, widely available, and associated with few minor complications, as seen in our series, which are typically easily manageable, such as pneumothorax and alveolar hemorrhage, and usually do not require additional procedures. Dendo et al. reported two cases of thoracic drainage in 168 fiducial placements, both of which were asymptomatic.^(9)^ In contrast, navigational tools face limitations, such as high cost, limited availability, and a steep learning curve, requiring considerable expertise and resources.^(10,12)^

Gilberto et al. described cases of cone-beam CT-guided preoperative localization of lung nodules in a hybrid room, using a hook wire marker and metallic microcoils. They stated that microcoils were preferred owing to their lower dislodgement rates. Furthermore, hybrid rooms, when available, may offer the additional benefit of lowering complication rates, such as dislodgement and pneumothorax.^(13)^

The conspicuous visibility of the metallic wires on the pulmonary surface during surgery is a testament to their usefulness. Specifically, the Somatex^®^ lung marker system offers additional advantages, such as the possibility of repositioning the coil-end of the wire during the procedure if necessary. Moreover, once positioned inside the lesion, there is a very low possibility of displacement because the spiral adheres more effectively to the lung nodule. Additionally, the material is already prepared for use, eliminating the preparation step and consequently reducing the procedure time.

Nonetheless, the judicious use of metallic markers, albeit necessitating fluoroscopy during surgery, results in mild complications with no substantive impact on subsequent VATS procedures.

A drawback of our study is its retrospective nature and the absence of a control group. Nonetheless, the findings support the device's utility, demonstrating excellent safety and a low complication rate.

## CONCLUSION

Preoperative CT-guided localization of small nonpalpable pulmonary lesions using a sophisticated spiral wire marker system has emerged as a safe and effective precursor to thoracic surgical intervention.
